# Immunity Dynamics of *Neisseria meningitidis* Serogroups ACYW from Birth and Following Vaccination

**DOI:** 10.3390/vaccines12111274

**Published:** 2024-11-13

**Authors:** Lilian Zeng, Yingyin Deng, Chumin Liang, Zixia Qian, Yueling Chen, Huifang Lin, Runyu Yuan, Pingping Zhou, Xue Zhuang, Ying Yang, Qi Zhu, Limei Sun, Jianfeng He, Jiufeng Sun

**Affiliations:** 1 Guangdong Provincial Institute of Public Health, Guangzhou 511430, China; zenglilian@yeah.net (L.Z.); lcm785169953@163.com (C.L.); colilam1226@163.com (H.L.); cecilia_yry@hotmail.com (R.Y.); zpp1015@foxmail.com (P.Z.); cher512364337@163.com (X.Z.); yang99063@126.com (Y.Y.); 2 School of Public Health, Sun Yat-sen University, Guangzhou 510080, China; dengyy66@mail2.sysu.edu.cn; 3 School of Public Health, Guangdong Pharmaceutical University, Guangzhou 510310, China; 13610257243@163.com (Z.Q.); 2112241063@stu.gdpu.edu.cn (Y.C.); 4 Guangdong Provincial Center for Disease Control and Prevention, Guangzhou 511430, China; zqraul7@163.com (Q.Z.); hjf@cdcp.org.cn (J.H.)

**Keywords:** *Neisseria meningitidis*, antibody levels, vaccines

## Abstract

**Background**: Serosurveillance of epidemic cerebrospinal meningitis (ECM) in healthy individuals is crucial for assessing disease risk and evaluating the effectiveness of vaccinations. However, this practical work is rare in China. **Methods**: We conducted cross-section serosurveillance in Guangzhou, Zhanjiang, and Heyuan in Guangdong Province, measuring Anti-Nm IgG with serogroups A, C, Y, and W, and analyzed the trends using a generalized additive model (GAM). **Results**: During 2019–2022, 7752 participants were included. The overall antibody positivity rate for serogroups A, C, Y, and W were 60.75%, 15.51%, 32.83%, and 14.56%, respectively. High Anti-Nm IgG was in children aged 0–5 and 5–10 years old. Geometric mean concentrations (GMCs) of Anti-Nm IgG were higher and correlated positively with vaccine doses compared with unvaccinated individuals. The GMC showed a consistent decrease trend in the vaccinated and a U-shaped curve in populations. The declined rates of GMC were 1.59 (95% CI: 1.03, 2.14) µg/mL, 1.65 (95% CI: 1.28, 2.03), 0.62 (95% CI: 0.22, 1.03), and 0.31 (95% CI: 0.08, 0.53) µg/mL per year for serogroups A, C, Y, and W, respectively. **Conclusions**: There were differences in antibody positivity rate and GMC for the four serogroups of ECM in the healthy individuals of Guangdong Province, with serogroup A showing the highest, and the demographic differences highlighted the high seroprevalence of *Neisseria meningitidis* in younger people. The variable prevalence rates among serogroups A, C, Y, and W and the observed decline in antibody titers underscore the need for adjustments in the immunization program targeting the meningococcal vaccine.

## 1. Introduction

*Neisseria meningitidis* (*N. meningitidis*), a Gram-negative diplococcus that can colonize the upper respiratory tract asymptomatically and may breach the mucosal barrier to cause epidemic cerebrospinal meningitis (ECM), is spread mainly by droplets of respiratory secretions from patients or asymptomatic carriers, especially during coughing and sneezing [1]. Despite the efficacy of vaccines in reducing the incidence of ECM, there are still nearly 1.2 million cases emerging globally each year [2,3]. People of all ages are susceptible to ECM, especially those under the age of 15 [4]. In China, a developing country with a population of 1.4 billion, the public health crisis posed by ECM is particularly acute. The case-fatality rate of ECM in China in 2021 was as high as 11.68%, and the most serious cases were in children under 1 year of age, with a case-fatality rate of more than 25% [5]. In addition, the financial burden is also heavy. A study in China showed that the average treatment cost of each ECM case was over CNY 73,000 [6], with direct medical costs and total economic spending accounting for 27.06% and 47.68% of the annual per capita disposable income of urban residents, respectively [7].

The identification of the serogroup of *N. meningitidis* is essential for prevention and control strategies. In the context of differences in the prevalence history of bacterial flora in various regions, more than 10 serogroups of *N. meningitidis* have been identified around the world, of which the most predominant causing ECM are serogroups A, B, C, W, Y, and X. In China, serogroup A was predominantly prevalent before 2000, followed by a serogroup C outbreak in Anhui Province in 2003 [8]. In 2006–2007, the first cases of serogroup W and serogroup X were reported in Fujian Province and Beijing Municipality, respectively [9,10]. Between 2015 and 2019, 296 laboratory-confirmed ECM cases were reported in China, with serogroups A, B, C, W, Y, and other ungrouped serogroups accounting for 4.73%, 36.15%, 22.97%, 6.08%, 1.69%, and 28.38%, respectively [11].

Vaccination is one of the most cost-effective prevention measures for ECM. Since the introduction of meningococcal polysaccharide vaccine group A (MPSV-A) and meningococcal polysaccharide vaccine groups A and C (MPSV-AC) into the immunization plan of China in 2007, the incidence of serogroups A and C showed a decreasing trend year by year in China [11,12,13]. For the prevention and control of diseases caused by other serogroups of *N. meningitidis*, the serogroup A, C, Y, and W meningococcal polysaccharide vaccine (MPSV-ACYW) was approved in China in 2008. However, the protective effect of the meningococcal polysaccharide vaccine (MPSV) is ultimately limited [14,15]. The serogroup A and C meningococcal polysaccharide conjugate vaccine (MPCV-AC) and the serogroup A, C, Y, and W meningococcal polysaccharide conjugate vaccine (MPCV-ACYW) were approved for marketing in 2006 and 2021, respectively, but are classified as Class II vaccines in China, requiring out-of-pocket expenses [16]. According to the National Immunization Program (2021 Edition) Vaccination Procedures and Instructions, children should receive one dose of MPSV-A at 6 months and at 9 months, or one dose of MPSV-AC at 3 and 6 years of age. The promotion of more vaccines into the national immunization program is important for the prevention and control of ECM.

Recent data indicate that sporadic cases and occasional outbreaks of ECM still occur frequently in Guangdong Province, with a notable increase in serogroups B, W, and Y, despite a declining trend in serogroups A and C [17,18,19]. Understanding the serotype pattern shift in healthy populations is essential for public authorities to timely assess the risk of ECM outbreaks. In this study, we analyze the survey results of serogroups A, C, Y, and W among healthy individuals in Guangdong Province from 2019 to 2022, evaluate the effects of vaccination, and quantify the trend of antibody titers decay over time. The findings will support the reform of vaccination programs in Guangdong and could be informative for other regions experiencing endemic ECM.

## 2. Methods

### 2.1. Sampling Design

**Figure 1:**
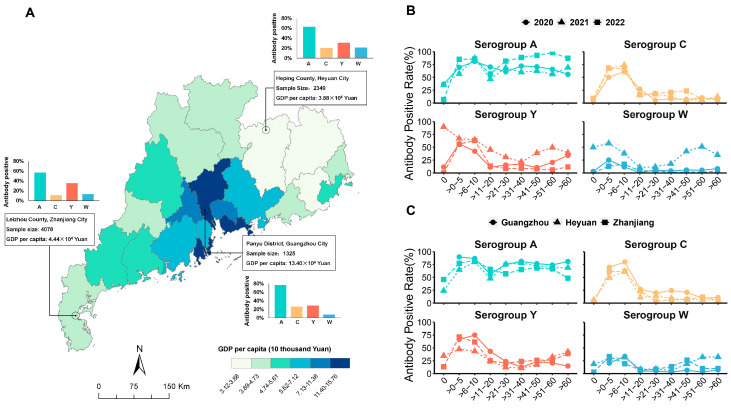
Regional distribution of the study sample and age distribution of antibody positivity for serogroups A, C, Y, and W. (**A**) The number of study samples and the antibody positivity rates in different serogroups (the background color is the spatial distribution of GDP per capita in 2020 for each city in Guangdong Province, China). (**B**) Antibody positivity rates for serogroups A, C, Y, and W in different age groups by years. (**C**) Antibody positivity rates for serogroups A, C, Y, and W in different age groups by cities.

A multi-stage stratified random sampling method was employed to select participants from Guangdong Province. Firstly, all 21 cities in the province were categorized into three economic strata. One city from each stratum was randomly chosen: Guangzhou from the high, Heyuan from the middle, and Zhanjiang from the low economic stratum. Second, one district or county was randomly selected in each sampled city: Panyu District from Guangzhou, Heping County from Heyuan, and Leizhou County from Zhanjiang (Figure 1A). Finally, in each district or county, a grade 2A or higher hospital was selected as the monitoring site, as grade 2A or higher hospitals serve the entire population and have a complete information system, which ensured representativeness of the sample and data collection. The sample size was calculated by the following formula:

$$n=\frac{Z_{\alpha /2}^{2}\left(1-P\right)}{\delta^{2}P}$$

where *n* denotes the sample size, the error probability *α* is 0.05 (two-sided) and the corresponding value of $Z_{\alpha /2}$ is 1.96, and the tolerance error *δ* is taken to be 0.05. *P* can be expressed in terms of antibody positivity rate in the population. Previous studies have shown that the antibody positivity rate of *N. meningitidis* in the healthy population is in the range of 70–90%, thus, we take the value of *P* as 70%. Based on the values of the above parameters, the minimum sample size of *n* = 658 was calculated. Considering the influence of non-response or other factors, we randomly included a sample of 1000 persons per hospital per year. In order to make the data from different hospitals comparable, a number of quality control measures were implemented, including (1) uniform training of investigators; (2) use of a uniform questionnaire; (3) use of the same specification and model of sample collection equipment; (4) use of the same inclusion criteria: participants must have lived in the area for at least 3 months; and (5) use of the same exclusion criteria: a. refusal to collect venous blood, b. persons with infectious diseases and immune deficiency or immunosuppressed patients. All participants included in the survey or their guardians signed informed consent forms.

### 2.2. Information Collection

The basic information of the participants was collected through questionnaires, including age, gender, residential address, and ID number. For children, guardians provided the information. ECM vaccination data, including dates, number of doses, and vaccine types, were retrieved from the Immunization Planning Information System (IPIS) of the Guangdong Provincial Center for Disease Control and Prevention by professional technicians.

### 2.3. Serum Sampling

Five milliliters of peripheral venous blood was collected from each participant. For newborns, umbilical cord blood was collected. All blood samples were centrifuged at 3000 rpm for 5 min, and the serum was transferred to a new freezer tube, labeled, and stored at −20 °C until analysis.

### 2.4. N. meningitidis IgG Antibody (Anti-Nm IgG) Test

Enzyme-linked immunosorbent assay (ELISA) was performed on all samples using serogroups A, C, Y, and W meningococcal polysaccharide IgG antibody kits (Lvzhu Biotech, Beijing, China) to test for the presence of Anti-Nm IgG. The experimental procedure was conducted strictly according to the kit’s manual instructions. The detailed procedure of the experimental operation is shown in the Supplementary Materials (supplementary methods). According to the manual, we adopted ≥2 µg/mL as the criterion for determining the positivity of Anti-Nm IgG in serogroups A, C, Y, and W.

### 2.5. Statistical Analysis

All qualitative data were expressed as constituent ratios or rates, and the differences between groups were tested using Pearson’s chi-square or Fisher’s exact probability method. Quantitative data were represented by means and standard deviations, and the differences between groups were tested by *t*-test or analysis of variance (ANOVA).

A generalized additive model (GAM) with a spline function was used to model the decay of antibody levels over time, with time since vaccination as the independent variable and antibody titer as the dependent variable. The GAM model allows the response variable to follow a variety of distributions from a family of exponential distributions, and the non-linear relationship between the data can be captured by choosing an appropriate link function [20]. The model equation is as follows:

$$E\left(Y\right)=\beta_{0}+s(time,df)$$

where *E*(*Y*) denotes the expected value of antibody titer, *β*_0_ denotes the intercept, *s* is a spline function, *time* represents the length of time between vaccination and sampling time, and *df* denotes the degree of freedom. The Akaike information criterion (AIC) of the model was used to evaluate the fitting effect of different *df*, and the minimum AIC model was selected as the final model. The model fits a non-linear curve between the length of time from vaccination to sampling time and antibody titer. If the curve appears to be non-linear, the threshold and time of antibody decay are reported; if the curve appears to be linear or approximately linear, the rate and time of antibody decay are reported. In addition, we also used this model to further fit the relationship between the antibody titers and the interval of birth time and sampling time.

All data were prepared and analyzed using R project software (version 4.2.1). The results of the statistical tests were two-sided with a value of *p* < 0.05 as statistical significance.

## 3. Results

### 3.1. Demographic Information of the Participants

From 2019 to 2022, a total of 7752 participants were included, of which 3391 (43.74%) were male and 4361 (56.26%) were female. The age distribution showed the largest group was individuals over 60 years old, accounting for 30.74% (n = 2383), followed by those aged 21–30 years old at 13.17% (n = 1021). Participants were distributed across three cities in Guangdong Province, including 1325 (17.09%) in Guangzhou, 2349 (30.30%) in Heyuan, and 4078 (52.61%) in Zhanjiang. In 2019–2022, the numbers of participants were 1563 (20.16%), 2615 (33.73%), 2085 (26.90%), and 1489 (19.21%), respectively.

### 3.2. The Seroprevalence of Anti-Nm IgG Among Participants

The Anti-Nm IgG positivity rates for serogroups A, C, Y, and W were 60.75%, 15.51%, 32.83%, and 14.56%, respectively (Table 1). Anti-Nm IgG positivity was higher in male than in female participants for serogroups C, Y, and W. The highest positivity rates across all serogroups were found in the age groups 0–5 and 5–10 years. By city, Guangzhou showed higher rates for serogroups A and C, Zhanjiang for serogroup Y, and Heyuan for serogroup W. In 2019–2022, Anti-Nm IgG positivity rates for serogroups A and C showed an increasing trend when compared with serogroups Y and W, with a fluctuating trend. The geometric mean concentrations (GMCs) of Anti-Nm IgG within the different serogroups showed the same distribution as the positivity rate (Table 1). Tests of statistical differences between groups are shown in Table S1. Except for gender, the Anti-Nm IgG positivity rate and GMC between the groups in terms of age, city, and year were the statistical differences.

The Anti-Nm IgG positivity rates for serogroups A, C, Y, and W in cord blood were 32.96%, 4.47%, 28.13%, and 13.76%, respectively. In addition, we also observed that the GMCs for serogroups A, C, Y, and W in > 0–1 year age in the unvaccinated groups were 4.19 (95% CI: 3.32, 5.28) µg/mL, 2.81 (95% CI: 1.73, 4.55) µg/mL, 4.82 (95% CI: 3.85, 6.05) µg/mL, and 2.93 (95% CI: 2.12, 4.06) µg/mL, respectively, which were slightly lower than the GMC of cord blood in the 0 year group, but the difference was statistically significant only for serogroup C (*p* < 0.01).

We also analyzed the age-related trends in Anti-Nm IgG positivity by years and cities (Figure 1B,C). The Anti-Nm IgG positivity rate in serogroups A and C showed a consistent age distribution pattern across the years, while it did not for serogroups Y and W in 2021. No differences within Guangzhou, Zhanjiang, and Heyuan between Anti-Nm IgG positivity rates and age for all serogroups were observed.

### 3.3. Vaccination and Seroprevalence of Anti-Nm IgG

**Figure 2:**
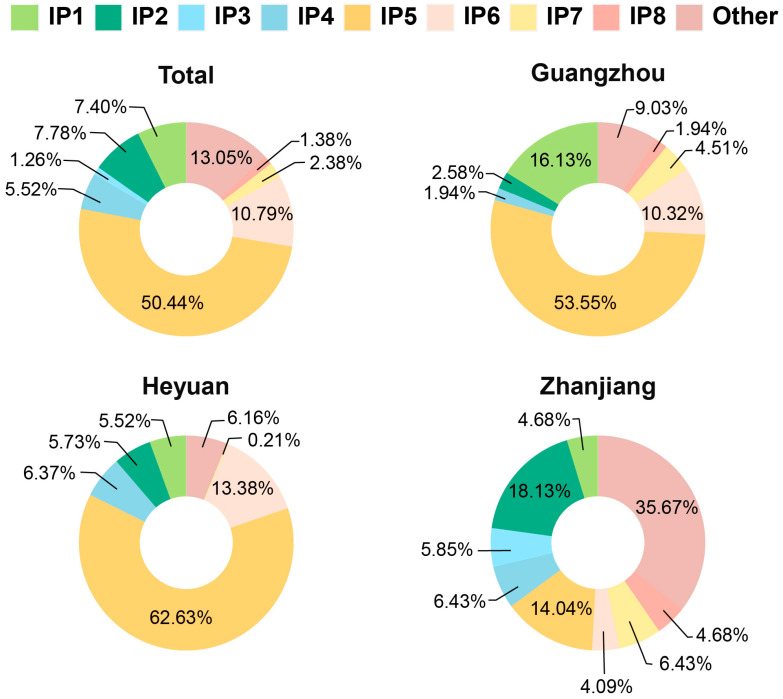
Distribution of immunization procedures (IP) in the vaccinated sample. (The details of each immunization procedure are shown in Table S4.)

Of the 7752 participants, 797 (10.22%) received at least one dose of *N. meningitidis* vaccine, while the remaining 6955 (89.78%) had no record of vaccination (Table S2). Among the vaccinees, there were mainly eight different immunization procedures (IP), with the combination of MPSV-A, MPSV-A and MPSV-AC being the most prevalent (50.44%), followed by the combination of MPSV-A, MPSV-A, MPSV-AC and MPSV-AC (10.79%). In the cities of Guangzhou and Heyuan, this same combination dominated. In contrast, Zhanjiang showed a more diverse distribution, with MPSV-AC (18.13%) being the most prevalent (Figure 2, Table S2).

**Figure 3:**
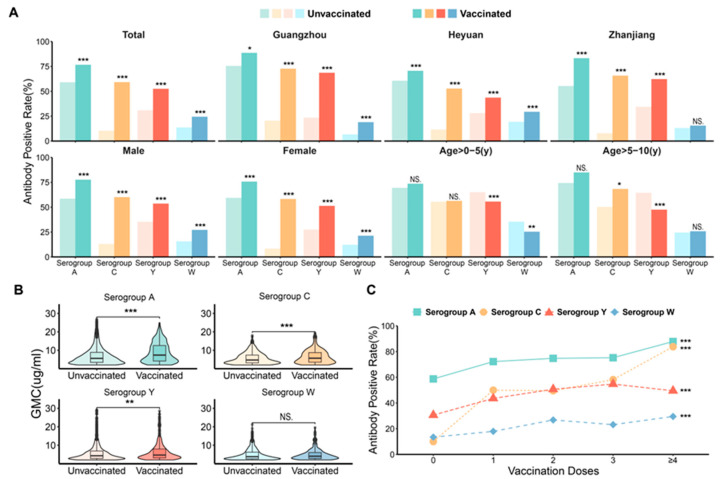
Antibody positivity rate and geometric mean concentrations (GMCs) for serogroups A, C, Y, and W in vaccinated and unvaccinated samples. (**A**) The antibody positivity rate of serogroups A, C, Y, and W in vaccinated and unvaccinated samples by city, sex, and age (*** *p*  <  0.001; ** *p*  <  0.01; * *p*  <  0.05; NS, not significant). (**B**)The geometric mean concentrations (GMCs) for serogroups A, C, Y, and W in vaccinated and unvaccinated samples (*** *p*  <  0.001; ** *p*  <  0.01; NS, not significant). (**C**) The antibody positivity rate of serogroups A, C, Y, and W in study samples with different vaccinated doses (*** *p* trend  <  0.001).

We further investigated the seropositivity and GMC of antibodies in both the vaccinated and unvaccinated populations. Overall, the Anti-Nm IgG positivity rate and GMC of serogroups A, C, Y, and W were higher in the vaccinated participants compared with the unvaccinated (Figure 3A,B). There was a significant trend of increasing antibody positivity as the number of doses increased (Figure 3C). Similar characteristics were found in the subgroups of gender and city. However, the age groups of >0–5 years and >5–10 years showed some deviations, particularly among the unvaccinated in serogroup Y, where Anti-Nm IgG positivity was higher than in the vaccinated group (Tables S3 and S4). The Anti-Nm IgG positivity rates and GMCs of the immunization procedures for different subgroups are displayed in Table S5.

Regarding the number of vaccine doses, the antibody positivity rate of serogroups A, C, Y, and W were higher in individuals who received booster immunizations (≥3 doses) compared with those with basic immunization (2 doses), and these rates increased with the number of doses administered (*p* < 0.001).

### 3.4. The Decay Tendency of Anti-Nm IgG

**Figure 4:**
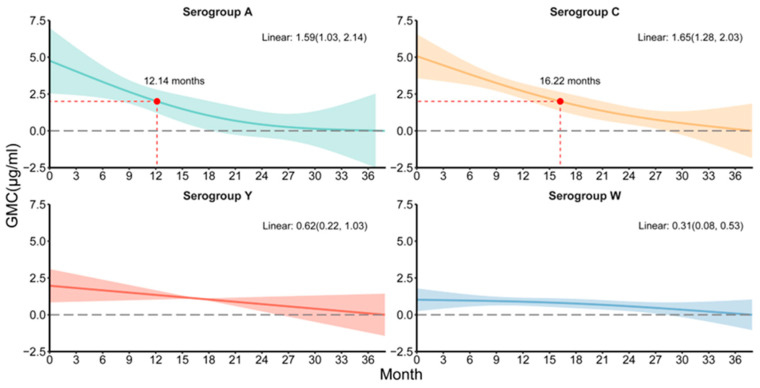
The decay tendency of Anti-Nm IgG for different serogroups (GMC: geometric mean concentration. Solid lines indicate model fitting associations, and shading indicates 95% confidence intervals. The red dot represents a GMC of 2 µg/mL).

Within the vaccinated participants, the curves of GMCs at vaccination-to-sampling time intervals showed a monotonically decreasing trend in GMC over time for serogroups A, C, Y, and W (Figure 4). After linearization, the rate of GMC decline was 1.59 (95% CI: 1.03, 2.14) µg/mL, 1.65 (95% CI: 1.28, 2.03) µg/mL, 0.62 (95% CI: 0.22, 1.03) µg/mL, and 0.31 (95% CI: 0.08, 0.53) µg/mL per year for serogroups A, C, Y, and W, respectively. According to the generalized additive models, the mean time of IgG antibody concentrations to fall below 2 µg/mL for serogroups A and C was 12.14 months and 16.22 months, respectively (Figure 4). The tendency of the GMC for different subgroups of the immunization procedures are displayed in Figure S1. Trends in GMC at birth-to-sampling time intervals for the vaccinated and unvaccinated populations were further fitted (Figure S2). For the vaccinated population, all four serogroups showed an approximately linear decreasing trend, but none of the decay rates of GMC were statistically significant after linearization. For the unvaccinated participants, the GMC for serogroup A showed a trend of flattening and then decreasing. In contrast, the tendency of GMC for serogroups C, Y, and W exhibited a U-shaped curve, with the lowest GMC at birth-to-sampling time intervals of 56.9, 45.8, and 44.3 years, respectively.

## 4. Discussion

Understanding the ECM antibody levels in healthy populations will facilitate the effectiveness evaluation of ECM vaccination. In this study, we determined the Anti-Nm IgG positivity and GMC of the four serogroups of ECM in healthy individuals and quantified the decreasing trend of antibody titers over time in Guangdong Province.

Overall, serogroup A had the highest Anti-Nm IgG positivity rate among all participants (60.75%), followed by serogroup Y (32.83%), and then serogroups C and W. This finding is consistent with the shift in ECM flora from serogroup A to serogroup C and the increasing prevalence of serogroup Y and W cases of epidemic encephalitis in recent years [11,21,22,23]. It also corresponds with the higher detection rates of *N. meningitidis* strains of serogroups C, Y, and W and the lower detection rate of serogroup A in Guangdong Province [18]. Compared with the archived studies in China, the positivity rates of Anti-Nm IgG for serogroups A and C in healthy individuals in Guangdong Province are lower than those reported in Jiangsu Province (72.13% and 54.69% in 2018–2020) [24], Luoyang City (69.05% and 46.67% in 2021) [25], and Shenzhen City (92.52% and 83.33% in 2009) [26]. The positive rate of serogroups Y and W were also lower than that in Tianjin, with 83.26% and 73.26% in 2017–2018 and 2020, respectively [27]. In addition, the Anti-Nm IgG positive rate of serogroup C (15.51%) was much lower than that of the national average (33.5%, 95% CI: 27.0–40.8%), suggesting a low endemic presence of serogroup C in Guangdong, which agrees with the epidemiological data in closing areas. Kang et al. [28] conducted a nationwide survey of human serum bactericidal antibody (HSBA) titers against *Neisseria meningitidis* serogroups A, C, W-135, and Y in people aged 11–50 years in the South Korea, which is similar in GDP size to Guangdong province, and found that the W-135 serogroup had a high percentage of people with protective antibodies with titers ≥ 8 (74%), while the A serogroup had the lowest percentage of people with protective antibodies. This differs from our findings in Guangdong Province, which may be related to local population density, hygiene conditions, and factors such as vaccination coverage, previous infections, or natural exposure of the population.

Subgroup analyses revealed significant differences in Anti-Nm IgG positivity and GMC across different age groups. Participants aged 0–5 years and 5–10 years exhibited higher positivity of Anti-Nm IgG and GMC in all four serogroups than those in other age groups. This coincided with the recommended immunization schedule [29]. In addition, we showed that Anti-Nm IgG was detectable in cord blood and that infants passively acquired protection from these maternal antibodies through the placenta. Compared with cord blood, the GMCs of serogroups A, C, Y, and W were decreased in the >0–1 year old and unvaccinated group. These data are similar to a study in the United Kingdom [30], in which seroprotection was more than 60% after birth, declined rapidly after 2 months, and averaged 25% after 4 months. A Chinese study also found that maternal antibodies reached ~30% by 6 months after birth [31]. These data suggest that the Anti-Nm IgG acquired through maternal to infant transmission has a limited duration. However, if the mother of the neonate has not received a booster dose or has not been previously vaccinated, the neonate may lack passive protection, and the risk of contracting *N. meningitidis* increases dramatically. As the meningococcal vaccine in the immunization program is administered after 6 months of age in China, infants under 6 months of age do not have access to timely protection, which is a concern that needs to be urgently considered by public health authorities. In addition, with the increase in age, the Anti-Nm IgG positive rate and GMC for all serogroups, except for serogroup A, decreased to some extent. This indicates the need for continued attention to meningococcal vaccination for children less than 6 years of age as well as increased public awareness and guidance on booster immunizations for adults. It also highlights the importance of enhanced surveillance of meningococcal disease in adolescents and adults.

We also observed spatial heterogeneity in Nm antibody positive rates in Guangdong Province, with higher overall antibody positive rates in Guangzhou, especially for serogroup A and serogroup C. This phenomenon may be related to several regional factors. Firstly, in economically developed cities, vaccination is well publicized and there is greater community acceptance of the vaccine, which may lead to higher vaccination and seroprevalence. Second, residents of economically developed cities may have been vaccinated earlier against *N. meningitidis*, with earlier vaccinations focusing more on serogroup A and serogroup C. This may be one of the reasons for the higher rate of positivity for groups A and C in Guangzhou. Thirdly, it may be related to the imbalance in the distribution of medical resources among cities. Developed cities can carry out more convenient vaccination services with adequate medical resources, while remote areas may have problems such as inadequate medical facilities and untimely vaccine supply, making it difficult for residents to receive timely and effective vaccination. Finally, population flows and densities in different cities also have an impact. Developed cities tend to have higher population densities and greater mobility, increasing the risk of disease transmission, which may lead the government to pay more attention to disease prevention and control and vaccination.

The positivity rate and GMC of Anti-Nm IgG were higher in serogroups A, C, Y, and W in vaccinated than in unvaccinated individuals, and the same characteristics were found in the gender and urban analyses. In particular, the study found no difference in Anti-Nm IgG positivity rates between the vaccinated and unvaccinated individuals in the 0–10 age group, which may be related to the potential hidden transmission of *N. meningitidis* between adults and children, as the bacteria can asymptomatically colonize the upper respiratory tract of adults and be transmitted through direct contact with respiratory secretions or droplets from asymptomatic carriers [32]. Additionally, it may be related to the immune persistence of MPSV-A with MPSV-AC. Previous studies have showed that the immune response elicited by MPSV in infants and children less than 2 years of age lasts only 1–2 years, and the protective antibodies produced by children ≥ 2 years of age last only 2–3 years [33,34]. In addition, the study also found that the positivity rate of Anti-Nm IgG was higher in those who completed booster immunization (≥3 doses) than those with basic immunization (≤2 doses), and the positivity rate increased with the number of doses of vaccine, which is consistent with the results of a previous study [35]. On further analysis, more than 89% of the individuals completing the basic immunization phase were children less than 10 years of age, all vaccinated with MPSV, which is less immunogenic. Studies have shown that the meningococcal polysaccharide conjugate vaccine (MPCV) can provide a more durable immunoprotective effect [36,37]. However, MPCV is still not included in the national immunization program in China. Therefore, optimizing the immunization procedures and strategies of meningococcal vaccines, and gradually promoting the involvement of MPCV and vaccines covering more *N. meningitidis* serogroups into the national immunization program, are of great significance for the prevention and control of ECM in China.

We also explored the trend in the decay of Anti-Nm IgG levels in vaccinated individuals over time. We found a nearly linear trend across the serogroups, with the mean annual decay rate varying between 0.22 and 1.65. Milou et al. [38] observed that the mean annual decay rate varied between 0.58 and 1.65 over a period of 1 to 5 years after vaccination, and the results of the present study are in general agreement with this. After an initial steep decline in antibody levels in the first year after vaccination, antibody levels slowly declined between 1 and 3 years post-booster [35]. We also found that serogroup C had the greatest average annual decline, which is consistent with the lower overall antibody positivity results for serogroup C monitored in this study. It is recommended that group C meningococcal vaccine booster immunization and group C meningitis surveillance should be promoted alongside continued meningococcal vaccination. In addition, we analyzed the decreasing trend of antibody levels from birth to sampling time in participants and found that in the unvaccinated population, the trend of antibody levels showed a non-linear pattern, with the lowest GMC occurring between 44.3 and 56.9 years of age. This emphasizes the importance of completing vaccination before this age. The influence of the age of priming was highlighted by other studies showing that primary vaccination at an older age induced longer persistence of antibodies [39,40,41,42,43]. In addition, strengthening vaccination for serogroups Y and W as well as recommending booster immunization for adults within a specific age group (especially before the age of 44.3 to 56.9 years) can yield multiple economic benefits. Firstly, it can alleviate the burden on medical resources by preventing hospitalization, treatment, and expenses related to complication management that are caused by infections. Secondly, it can reduce the loss of working hours, thereby enhancing labor productivity.

This study has several limitations that must be declared. First, regarding the selection of detection methods, the enzyme-linked immunosorbent assay (ELISA) used in this study measures the total IgG level of *Neisseria meningitidis* (Nm). However, the bactericidal activity of antibodies is related to only certain subclasses of IgG. Therefore, compared with the “gold standard” serum bactericidal assay, the accuracy of the research results may have a gap. But according to a previous study, the results measured by ELISA are similar to those of the bactericidal assay [44]. Therefore, the results of this study still have great reference value. Second, this study detected the antibody levels of only the Nm serogroups A, C, Y, and W. The situation of Nm serogroup B, which has been on the rise in Guangdong Province in recent years, was ignored. Further research is needed. Third, due to the unavailability of data, the GAM used in this study did not include other variables that change over time, such as natural exposure history, so the results may be biased. Finally, this study collected data from only three cities in Guangdong Province, and despite the use of stratified sampling, the possibility of under-representation cannot be completely eliminated. Moreover, the survey did not collect information on the participants’ economic status, living habits, and natural exposure history, so caution is needed when extrapolating the research conclusions.

## 5. Conclusions

This study highlights a high seroprevalence of *Neisseria meningitidis* in younger individuals in Guangdong. We determined the immunity dynamics of *N. meningitidis* serogroups A, C, Y, and W in healthy individuals of Guangdong Province from 2019 to 2022. The Anti-Nm IgG positivity rates for serogroups A, C, Y, and W were 60.75%, 15.51%, 32.83%, and 14.56%, respectively. Vaccination efforts should be enhanced for serogroups Y and W, and booster vaccinations are highly recommended in adults before age 44.3 to 56.9 years.

## Figures and Tables

**Table 1:** Number of samples tested, antibody positivity rate, and geometric mean concentration (GMC) for serogroups A, C, Y, and W.

Group	Serogroup A			Serogroup C			Serogroup Y			Serogroup W		
	Test	Antibody Positive (n, %)	GMC (μg/mL)	Test	Antibody Positive (n, %)	GMC (μg/mL)	Test	Antibody Positive (n, %)	GMC (μg/mL)	Test	Antibody Positive (n, %)	GMC (μg/mL)
**Total**	4965	3016 (60.75)	5.89 (5.76, 6.02)	4965	770 (15.51)	5.18 (4.98, 5.39)	7731	2538 (32.83)	4.80 (4.68, 4.91)	7612	1108 (14.56)	4.22 (4.08, 4.35)
**Sex**												
Male	2083	1267 (60.83)	5.98 (5.76, 6.20)	2083	397 (19.06)	5.33 (5.05, 5.64)	3385	1263 (37.31)	5.04 (4.86, 5.22)	3322	560 (16.86)	4.32 (4.12, 4.53)
Female	2882	1749 (60.69)	5.82 (5.66, 5.99)	2882	373 (12.94)	5.02 (4.75, 5.30)	4346	1275 (29.34)	4.57 (4.42, 4.72)	4290	548 (12.77)	4.11 (3.93, 4.30)
**Age (Y)**												
0	358	118 (32.96)	4.50 (4.11, 4.93)	358	16 (4.47)	3.80 (3.12, 4.63)	487	137 (28.13)	4.83 (4.48, 5.22)	487	67 (13.76)	3.25 (2.95, 3.57)
>0–5	566	408 (72.08)	7.01 (6.58, 7.48)	566	316 (55.83)	5.62 (5.29, 5.97)	929	548 (58.99)	5.72 (5.40, 6.05)	929	269 (28.96)	4.50 (4.21, 4.80)
>5–10	194	159 (81.96)	7.53 (6.80, 8.34)	194	123 (63.4)	5.84 (5.32, 6.43)	357	198 (55.46)	5.32 (4.84, 5.85)	357	89 (24.93)	3.85 (3.45, 4.29)
>10–20	302	170 (56.29)	5.98 (5.45, 6.56)	302	59 (19.54)	5.44 (4.69, 6.31)	587	175 (29.81)	4.23 (3.90, 4.59)	587	41 (6.98)	3.77 (3.22, 4.40)
>20–30	716	453 (63.27)	5.94 (5.61, 6.30)	716	59 (8.24)	5.19 (4.49, 6.00)	1017	204 (20.06)	4.23 (3.96, 4.51)	1017	76 (7.47)	3.63 (3.25, 4.05)
>30–40	543	387 (71.27)	5.92 (5.56, 6.30)	543	53 (9.76)	4.54 (3.93, 5.25)	837	147 (17.56)	3.90 (3.57, 4.27)	837	78 (9.32)	3.92 (3.45, 4.46)
>40–50	324	232 (71.6)	7.17 (6.64, 7.74)	324	29 (8.95)	4.51 (3.69, 5.52)	567	122 (21.52)	4.22 (3.78, 4.72)	565	111 (19.65)	3.82 (3.45, 4.23)
>50–60	344	235 (68.31)	6.16 (5.66, 6.70)	344	31 (9.01)	3.50 (2.99, 4.09)	573	162 (28.27)	4.36 (4.00, 4.74)	546	104 (19.05)	6.20 (5.55, 6.93)
>60	1618	854 (52.78)	4.97 (4.78, 5.18)	1618	84 (5.19)	4.30 (3.86, 4.79)	2377	845 (35.55)	4.75 (4.56, 4.96)	2287	273 (11.94)	4.23 (3.97, 4.50)
**City**												
Guangzhou	578	444 (76.82)	5.02 (4.88, 5.15)	578	153 (26.47)	4.98 (4.70, 5.28)	1317	376 (28.55)	5.11 (4.93, 5.28)	1317	103 (7.82)	4.09 (3.89, 4.30)
Heyuan	1472	920 (62.5)	6.30 (6.05, 6.57)	1472	298 (20.24)	4.76 (4.49, 5.04)	2339	723 (30.91)	4.68 (4.49, 4.88)	2339	493 (21.08)	4.62 (4.41, 4.84)
Zhanjiang	2915	1652 (56.67)	9.28 (8.71, 9.88)	2915	319 (10.94)	6.63 (6.00, 7.32)	4075	1439 (35.31)	3.96 (3.78, 4.15)	3956	512 (12.94)	3.15 (2.96, 3.36)
**Year**												
2019	1156	553 (47.84)	4.18 (4.01, 4.35)	1156	34 (2.94)	4.83 (4.08, 5.71)	1561	568 (36.39)	4.97 (4.73, 5.23)	1560	171 (10.96)	3.99 (3.66, 4.36)
2020	2568	1602 (62.38)	5.34 (5.21, 5.48)	2568	442 (17.21)	4.78 (4.56, 5.02)	2607	713 (27.35)	5.39 (5.12, 5.67)	2607	231 (8.86)	4.99 (4.62, 5.39)
2021	610	363 (59.51)	6.60 (6.16, 7.07)	610	119 (19.51)	4.76 (4.35, 5.21)	2082	954 (45.82)	4.64 (4.47, 4.82)	1964	628 (31.98)	4.18 (4.01, 4.35)
2022	631	498 (78.92)	10.84 (10.25, 11.45)	631	175 (27.73)	6.80 (6.22, 7.44)	1481	303 (20.46)	3.79 (3.61, 3.97)	1481	78 (5.27)	3.10 (2.90, 3.33)

For GMC, 95% confidence intervals in brackets.

## Data Availability

The raw data were generated by the Guangdong Provincial Center for Disease Control and Prevention. Derived data supporting the results of this study are available from the corresponding authors on request for non-commercial use only and are subject to a signed data use agreement. The R codes supporting the results of this study are available from the corresponding author upon request.

## Supplementary Materials

The following supporting information can be downloaded at: https://www.mdpi.com/article/10.3390/vaccines12111274/s1, Figure S1: The geometric mean concentrations (GMC) of serogroups A, C, Y and W in different immunization procedures (IP); Figure S2: Trends in geometric mean concentrations (GMC) of serogroups A, C, Y and W over time from birth in vaccinated and unvaccinated sample; Table S1: Statistical tests of antibody positivity and geometric mean concentrations (GMC) of serogroups A, C, Y and W by sex, age, city and year; Table S2: Distribution of immunization procedures (IP) in vaccination samples (n, %); Table S3: The antibody positivity rate of serogroups A, C, Y and W in vaccinated and unvaccinated samples; Table S4: Statistical test for serogroup A, C, Y and W antibody positivity in vaccinated and unvaccinated samples; Table S5: Number of samples tested, antibody positivity rate and geometric mean concentration (GMC) for serotypes A, C, Y and W by immunization procedures.
