# Supplementary material for: Immunity Dynamics of Neisseria meningitidis Serogroups ACYW from Birth and Following Vaccination
Source: Vaccines (Basel). 2024 Nov 13;12(11):1274. doi: 10.3390/vaccines12111274 (PMC11599020; doi:10.3390/vaccines12111274)
Supplement: Supplementary file 1 [file vaccines-12-01274-s001.zip › vaccines-3260813-supplementary.pdf]

Supplementary materials

Immunity Dynamics of *Neisseria meningitidis* Serogroups ACYW from Birth and Following Vaccination

CONTENT

1. Supplementary Methods: Nm IgG Antibody (Anti-Nm IgG) Test..3

2. Supplementary Tables and Figures 4

Table S1. Statistical tests of antibody positivity and geometric mean concentrations (GMC) of serogroups A, C, Y and W by sex, age, city and year..4

Table S2. Distribution of immunization procedures (IP) in vaccination samples (n, %) 5

Table S3. The antibody positivity rate of serogroups A, C, Y and W in vaccinated and unvaccinated samples...6

Table S4. Statistical test for serogroup A, C, Y and W antibody positivity in vaccinated and unvaccinated samples...7

Table S5. Number of samples tested, antibody positivity rate and geometric mean concentration (GMC) for serotypes A, C, Y and W by immunization procedures 8

Figure S1. The geometric mean concentrations (GMC) of serogroups A, C, Y and W in different immunization procedures (IP)..9

Figure S2. Trends in geometric mean concentrations (GMC) of serogroups A, C, Y and W over time from birth in vaccinated and unvaccinated sample 10

1. Supplementary Methods: **Nm IgG Antibody (Anti-Nm IgG) Test**

The standard serum with anti-Nm IgG in PBS was the positive control and the negative control was PBS without anti-Nm IgG. The serum samples were initially diluted at a ratio of 1:100 and then 100 μL of dilution was added to the determination plate for 1 h incubation at 37°C. After incubation, the plate was washed with an automatic plate washing machine (BioTek 405 TS, Winooski, VT, USA) using lotion buffer solution, repeated four times. This was followed by 100 μL IgG enzyme-labeled antibodies being added to each well and then incubated at 37°Cfor 30 min. After five times washing step, 100 μL of each equal volumes of mixed A and B color developers was added to each well and incubated again at 37°Cfor 10 min. Finally, 50 μL of termination solution was added. All the ELISA results were determined using an enzyme-labeled instrument (BioTek Epoch, Winooski, VT, USA), and the data were presented as the OD (optical density) value at the wavelength λ= 450 nm. The test was established when positive control A450＞0.50 and negative control A450＜0.20. Each measurement should be performed as a standard curve, with the standard antibody concentration as the abscissa and the absorbance value as the ordinate as the linear regression equation, and the equation is $y = a + bx$. Substituting the absorbance value (y) of the sample to be tested into the equation calculates the antibody concentration values (x,ug/ml) of the serum sample at different dilutions. The mean serum antibody concentration values is calculated by multiplying the corresponding sample dilution. The absorbance value of the sample beyond the range of the linear regression equation must not be substituted into the equation to calculate the antibody content.

2. Supplementary Tables and Figures

Table S1. Statistical tests of antibody positivity and geometric mean concentrations (GMC) of serogroups A, C, Y and W by sex, age, city and year.

Group	Serogroup A		Serogroup C		Serogroup Y		Serogroup W	
	χ^2/t/F	*P*	χ^2/t/F	*P*	χ^2/t/F	*P*	χ^2/t/F	*P*
**Antibody positive rates**								
Sex	0.005	0.94	34.1	<0.001	54.5	<0.001	24.7	<0.001
Age	280.0	<0.001	1274.6	<0.001	588.5	<0.001	305.9	<0.001
City	84.8	<0.001	124.6	<0.001	26.2	<0.001	136.4	<0.001
Year	171.5	<0.001	224.4	<0.001	306.6	<0.001	666.3	<0.001
**Geometric mean concentrations (GMC)**								
Sex	1.3	0.26	2.3	0.13	15.5	<0.01	2.2	0.14
Age	19.2	<0.001	5.8	<0.001	10.6	<0.001	10.4	<0.001
City	194.7	<0.001	20.8	<0.001	26.3	<0.001	22.9	<0.001
Year	288.3	<0.001	19.8	<0.001	25.4	<0.001	16.6	<0.001

For antibody positivity, chi-square test was used. For GMC, t-test was used for statistical tests for sex and ANOVA test for age and city.

Table S2. Distribution of immunization procedures (IP) in vaccination samples (n, %).

Immunization procedures	Guangzhou	Heyuan	Zhanjiang	Total
IP1: MPSV-A& MPSV-A	25 (16.13)	26 (5.52)	8 (4.68)	59 (7.40)
IP2: MPSV-AC	4 (2.58)	27 (5.73)	31 (18.13)	62 (7.78)
IP3: MPSV-ACYW	0(0.00)	0(0.00)	10 (5.85)	10 (5.85)
IP4: MPSV-A&MPSV-AC	3 (1.94)	30 (6.37)	11 (6.43)	44 (5.52)
IP5: MPSV-A& MPSV-A&MPSV-AC	83 (53.55)	295 (62.63)	24 (14.04)	402 (50.44)
IP6: MPSV-A&MPSV-A&MPSV-AC& MPSV-AC	16 (10.32)	63 (13.38)	7 (4.09)	86 (10.79)
IP7: MPSV-A& MPSV-A& MPSV-ACYW	7 (4.52)	1 (0.21)	11 (6.43)	19 (2.38)
IP8: MPCV-AC& MPCV-AC& MPSV-ACYW	3 (1.94)	0(0.00)	8 (4.68)	11 (1.38)
Other	14 (9.03)	29(6.16)	61 (35.67)	104 (13.05)
Total	155 (100.00)	471 (100.00)	171 (100.00)	797 (100.00)

Table S3. The antibody positivity rate of serogroups A, C, Y and W in vaccinated and unvaccinated samples.

Group	Serogroup A		Serogroup C		Serogroup Y		Serogroup W	
	**vaccinated**	**unvaccinated**	**vaccinated**	**unvaccinated**	**vaccinated**	**unvaccinated**	**vaccinated**	**unvaccinated**
**Total**	428 (76.43)	2588 (58.75)	330 (58.93)	440 (9.99)	416 (52.26)	2122 (30.6)	192 (24.12)	916 (13.44)
**Sex**								
Male	216 (77.42)	1051 (58.26)	167 (59.86)	230 (12.75)	222 (53.37)	1041 (35.06)	112 (26.92)	448 (15.42)
Female	212 (75.44)	1537 (59.09)	163 (58.01)	210 (8.07)	194 (51.05)	1081 (27.26)	80 (21.05)	468 (11.97)
**Age(Y)**								
0	/	118 (32.96)	/	16 (4.47)	/	137 (28.13)	/	67 (13.76)
>0-5	289 (73.35)	119 (69.19)	221 (56.09)	95 (55.23)	318 (55.4)	230 (64.79)	144 (25.09)	125 (35.21)
>5-10	122 (84.72)	37 (74)	98 (68.06)	25 (50)	87 (47.28)	111 (64.16)	47 (25.54)	42 (24.28)
>10-20	14 (77.78)	156 (54.93)	10 (55.56)	49 (17.25)	10 (29.41)	165 (29.84)	1 (2.94)	40 (7.23)
>20-30	2 (100)	451 (63.17)	0 (0)	59 (8.26)	1 (50)	203 (20)	0 (0)	76 (7.49)
>30-40	0 (0)	387 (71.4)	0 (0)	53 (9.78)	0 (0)	147 (17.58)	0 (0)	78 (9.33)
>40-50	1 (100)	231 (71.52)	1 (100)	28 (8.67)	0 (0)	122 (21.55)	0 (0)	111 (19.68)
>50-60	/	235 (68.31)	/	31 (9.01)	/	162 (28.27)	/	104 (19.05)
>60	/	854 (52.78)	/	84 (5.19)	/	845 (35.55)	/	273 (11.94)
**City**								
Guangzhou	61 (88.41)	383 (75.25)	50 (72.46)	103 (20.24)	106 (68.39)	270 (23.24)	29 (18.71)	74 (6.37)
Heyuan	225 (70.31)	695 (60.33)	168 (52.5)	130 (11.28)	204 (43.4)	519 (27.77)	137 (29.15)	356 (19.05)
Zhanjiang	142 (83.04)	1510 (55.03)	112 (65.5)	207 (7.54)	106 (61.99)	1333 (34.14)	26 (15.2)	486 (12.84)

Table S4. Statistical test for serogroup A, C, Y and W antibody positivity in vaccinated and unvaccinated samples.

	**Serogroup A**		**Serogroup C**		**Serogroup Y**		**Serogroup W**	
	χ^2	*P*	χ^2	*P*	χ^2	*P*	χ^2	*P*
**Total**	64.37	<0.001	904.42	<0.001	150.98	<0.001	64.56	<0.001
**Sex**								
Male	36.43	<0.001	344.53	<0.001	51.48	<0.001	33.60	<0.001
Female	27.74	<0.001	556.77	<0.001	93.58	<0.001	24.84	<0.001
**Age(Y)**								
>0-5	0.84	0.361	0.01	0.923	7.61	0.006	10.44	0.001
>5-10	2.21	0.137	4.47	0.035	9.61	0.002	0.02	0.878
**City**								
Guangzhou	5.19	0.023	82.49	<0.001	134.47	<0.001	27.21	<0.001
Heyuan	10.23	0.001	260.93	<0.001	42.26	<0.001	22.43	<0.001
Zhanjiang	50.30	<0.001	548.80	<0.001	54.39	<0.001	0.62	0.432

Table S5. Number of samples tested, antibody positivity rate and geometric mean concentration (GMC) for serotypes A, C, Y and W by immunization procedures.

Group	Serogroup A			Serogroup C			Serogroup Y			Serogroup W		
	Test (n, %)	Antibody positive(n, %)	GMC (μg/ml)	Test(n, %)	Antibody positive(n, %)	GMC (μg/ml)	Test(n, %)	Antibody positive(n, %)	GMC (μg/ml)	Test(n, %)	Antibody positive(n, %)	GMC (μg/ml)
IP1	39	25 (64.1)	8.01 (6.06, 10.58)	39	12 (30.77)	5.22 (3.78, 7.21)	59	32 (54.24)	5.68 (4.63, 6.97)	59	20 (33.9)	3.64 (2.95, 4.49)
IP2	58	42 (72.41)	5.48 (4.46, 6.73)	58	31 (53.45)	4.97 (4.20, 5.89)	62	23 (37.10)	6.30 (4.53, 8.78)	62	11 (17.74)	5.25 (3.51, 7.87)
IP3	10	8 (80.00)	7.48 (4.50, 12.43)	10	5 (50.00)	6.58 (3.81, 11.37)	10	10 (100.00)	8.81 (5.18, 14.97)	10	3 (30.00)	6.49 (0.96, 43.60)
IP4	34	29 (85.29)	7.03 (5.50, 8.98)	34	19 (55.88)	5.33 (4.27, 6.65)	44	14 (31.82)	5.44 (3.46, 8.57)	44	11 (25.00)	4.47 (2.91, 6.87)
IP5	248	178 (71.77)	6.53 (5.90, 7.22)	248	136 (54.84)	5.46 (4.91, 6.08)	402	204 (50.75)	4.79 (4.40, 5.21)	402	95 (23.63)	4.36 (3.94, 4.82)
IP6	58	53 (91.38)	7.89 (6.72, 9.27)	58	50 (86.21)	6.21 (5.47, 7.04)	85	41 (48.24)	5.11 (4.20, 6.22)	85	24 (28.24)	4.06 (3.23, 5.12)
IP7	17	13 (76.47)	9.47 (6.81, 13.16)	17	10 (58.82)	6.95 (4.02, 12.03)	19	17 (89.47)	5.91 (4.07, 8.60)	19	4 (21.05)	6.23 (1.12, 34.81)
IP8	10	9 (90.00)	8.03 (4.83, 13.37)	10	6 (60.00)	5.44 (2.22, 13.29)	11	9 (81.82)	5.91 (4.07, 8.58)	11	3 (27.27)	3.70 (1.25, 10.99)
Other	86	71 (82.56)	8.33 (7.18, 9.67)	86	61 (70.93)	5.83 (5.13, 6.62)	104	66 (63.46)	5.58 (4.72, 6.59)	104	21 (20.19)	4.68 (3.76, 5.82)

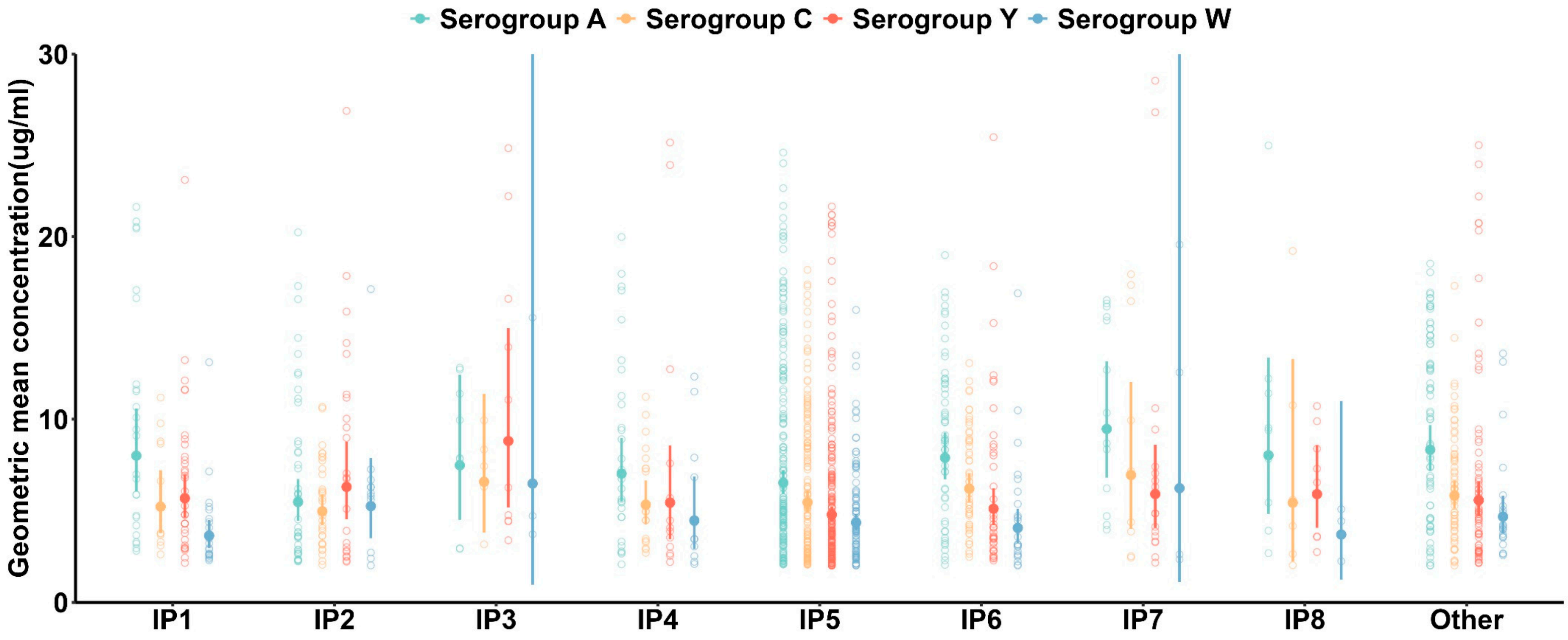

Figure S1. The geometric mean concentrations (GMC) of serogroups A, C, Y and W in different immunization procedures (IP).

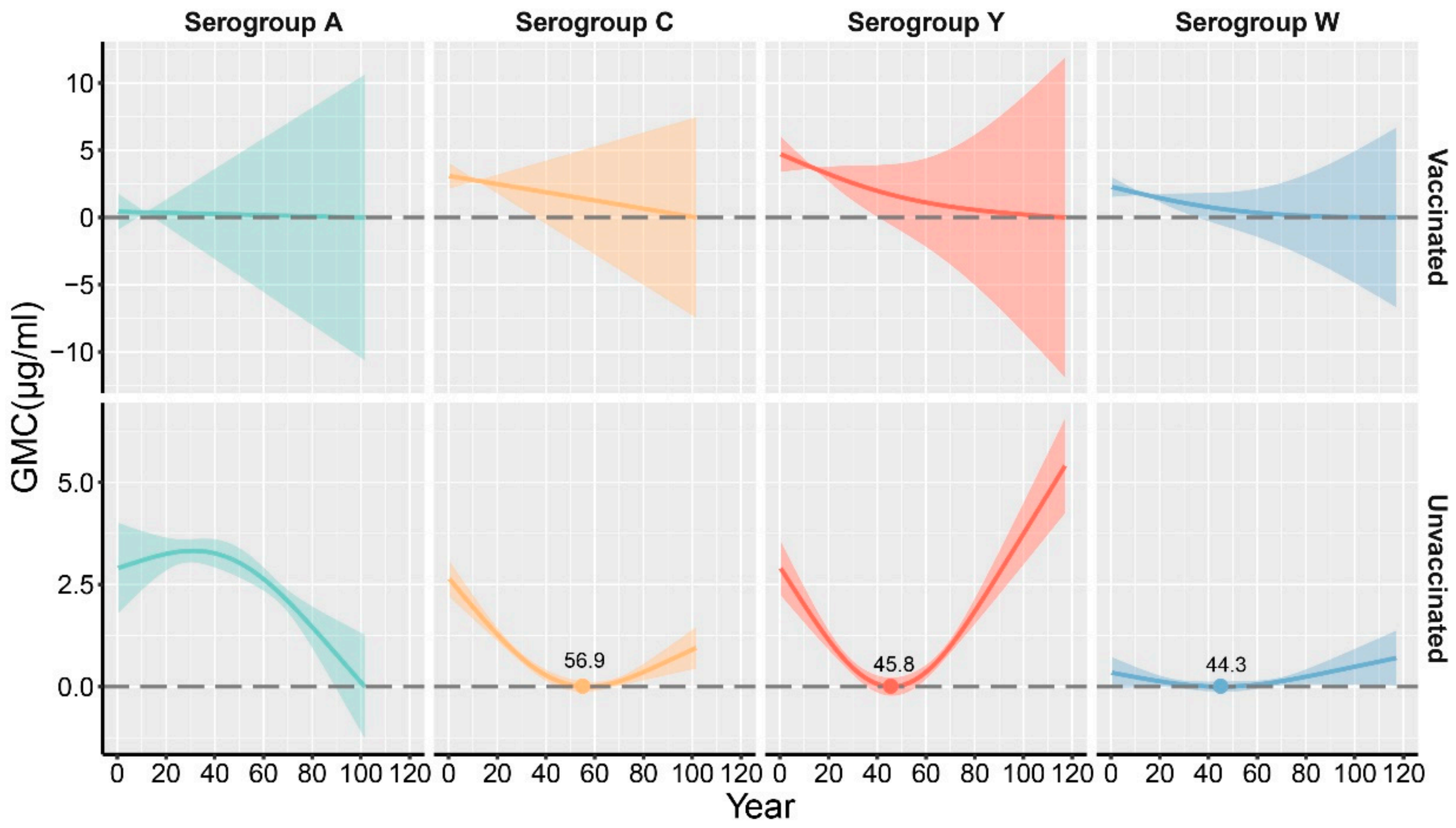

Figure S2. Trends in geometric mean concentrations (GMC) of serogroups A, C, Y and W over time from birth in vaccinated and unvaccinated sample.
